# What needs to be addressed in caring for people living with dementia? A multi-faceted evidence on financial, psychological, and physical health issues in dementia care

**DOI:** 10.3389/frdem.2026.1790741

**Published:** 2026-05-01

**Authors:** Ben Gould, Smruti Bulsari, Mariachiara Di Cesare

**Affiliations:** 1 Institute of Public Health and Wellbeing, University of Essex, Colchester, United Kingdom; 2 NIHR Applied Research Collaboration (ARC), Cambridge, United Kingdom

**Keywords:** Alzheimer’s disease, care, carer, dementia, financial implications, living with dementia, physical health impacts, psychological implications

## Abstract

**Background:**

Dementia is a progressive neurodegenerative condition with 60.2 million cases worldwide. The disease has financial, psychological, and physical health implications for people affected and their carers. There is a dearth of literature exploring financial issues experienced by carers and persons living with dementia beyond the loss of income, while psychological implications have been studied in silos, and physical health implications have mostly focused on sleep deprivation. Our study aims to provide multi-faceted evidence on all three issues experienced by persons living with dementia and their carers.

**Methods:**

Semi-structured interviews, congruent with the social constructivist framework, were administered, recorded on Microsoft Teams, and transcribed for eight carers, four persons living with dementia excepts, and one participant who was both a carer and a person living with dementia. Participants were recruited using a purposive sampling approach through the local community-led friendly cafe groups in Essex. Transcripts were anonymised, and thematic analysis was undertaken.

**Results:**

Responses of the carers revealed concerns about the future financial situation, the expenses on respite care, and replacing the household equipment. Psychological implications for carers included a change in role from partner to carer, resulting in loneliness, loss of friends, coping with the deterioration in behaviour of the person living with dementia except, and relentlessness of care. One major physical health implication identified was exhaustion and the need to extend physical support, such as lifting the person living with dementia from a fall. Persons living with dementia hardly expressed concerns on the financial implications, though they narrated denial of diagnosis, feeling of loss of independence, and the changes in behaviour associated with dementia, among the psychological implications. The physical implications of the persons living with dementia largely included physical deterioration. Other issues, including feeling at loss of access to information, post-diagnostic medical support, and designs of public toilets, were also elicited from the interviews.

**Conclusion:**

Given the interdependent nature of the issues experienced by persons living with dementia and their carers, an integrated approach to post-diagnostic support for information dissemination, counselling, and training for carers could help reduce both the physical and mental burden of dementia on patients and carers.

## 1 Introduction

Dementia and Alzheimer’s Disease are the leading causes of disability, dependency, and death globally, with an estimated worldwide dementia prevalence of 60.2 million people in 2023 [Institute for Health Metrics and Evaluation (IHME), 2025]. In the United Kingdom alone, the number of people living with dementia has now reached a million [Institute for Health Metrics and Evaluation (IHME), 2025], and dementia accounts for 11.4% of the total deaths in England and Wales (Office for National Statistics, 2023). It is estimated that of all diagnoses of dementia, 7.5% occur below the age of 65 years, commonly known as early-onset dementia (Carter, 2022).

Dementia is a chronic and progressive neurodegenerative condition characterised by cognitive decline and functional motor impairment; while it disproportionately affects those with previous genetic susceptibility (Qiu et al., 2009; Tariq and Barber, 2018). Multiple risk factors have been found to increase the probability of developing dementia including hypertension, hypercholesterolemia, obesity, smoking, heavy alcohol consumption, depression, hearing loss among others (Livingston et al., 2024; Tariq and Barber, 2018). Living with dementia not only has financial, psychological, and physical health implications on the persons living with dementia but also on the family members caring for them (Lindeza et al., 2024; Nandi et al., 2022).

The cognitive decline associated with dementia leads a person living with dementia to become dependent on either a family or a professional carer for their instrumental activities of daily living (IADL), such as managing finances, shopping, and cooking, and as the condition progresses, for activities of daily living (ADL), such as bathing, dressing, and eating (Raimo et al., 2024; Libon et al., 2023). Literature on the financial implications of dementia has largely focused on the economic burden of caring for dementia and its impact on the government (Bruno et al., 2018; Edwards et al., 2024; Lastuka et al., 2024) in different European countries. In the UK alone, the financial cost of dementia is expected to reach £90 billion by 2040, with estimates suggesting that 50% is associated with unpaid care (Alzheimer’s Society and Farrar, 2024). The economic burden of dementia extends beyond formal healthcare systems to affect informal (family) carers and family members (beyond immediate carers) to a great extent (Hu et al., 2024; Leniz et al., 2022; Nandi et al., 2022). The cost of informal care includes foregone wages by either giving up work totally or early retirement, and often in the form of reduced working hours, for both the person living with dementia and their carers (Nandi et al., 2024), termed as ‘opportunity cost’ in economics terminology. Informal care comprises many components over and above the opportunity cost of forgone work, including the cost of restructuring the house to support the person living with dementia and purchasing special equipment to support their ADL and IADL, and respite care.

Financial burden is often compounded by the emotional and physical toll of caregiving, creating a multifaceted impact on carers’ wellbeing (Steinsheim et al., 2023). Neuropsychiatric symptoms, such as depression, anxiety, irritability, euphoria, agitation, aggression, apathy, and disinhibition, are experienced by approximately 90% of persons living with dementia, and are found to strongly impact the quality of life for the person living with dementia and add burden to the carer (Banerjee et al., 2006; Borsje et al., 2018); they often precipitate transition to institutional care (Tan et al., 2005).

Physical health of the carer is affected by the relentlessness associated with care. Concurrently, the informal carer also ages alongside the progression of the condition. This implies an increased care burden with increasing age. Some carers themselves might be living with long-term health conditions or might have undergone an accident or surgery. Studies are exploring the effect of sleep deprivation experienced by the carers on their physical and mental health, as well as on their quality of caregiving (Cho et al., 2024; Jiwon et al., 2025; Mao et al., 2025).

There is limited literature exploring the financial implications of living with dementia from the perspectives of both, the persons living with dementia as well as their carers. Moreover, the studies exploring psychological implications are focused on the change in behaviour of the person living with dementia as an outcome of dementia. Fewer studies are exploring the psychological implications of living with dementia on carers. Similarly, fewer studies are exploring the implications of caring for the persons living with dementia on the physical health of the carer, and they examine the mediation of sleep deprivation because of caring responsibilities.

This study highlights the untapped in dementia care research by giving a multi-faceted evidence of financial, psychological, and physical health implications experienced by both the persons living with dementia and their carers, emphasising the implications on care. The objectives of this study are as follows:

1 To develop an understanding of the costs absorbed by families of the persons living with dementia.
2 To understand the emotions and psychological impact of the diagnosis, as well as living with the progressive nature of dementia, on the persons living with dementia and their carers.
3 To understand the physical health implications of caring for persons living with dementia on carers, beyond sleep deprivation.

## 2 Methods and materials

Research questions were identified based on existing literature on financial, psychological, and physical distress experienced by people living with dementia and their carers, and the University of Essex’s ethics approval was sought before participant recruitment (ETH2425-0291).

### 2.1 Population

The target population for this study was people living with dementia aged 60 years and above at the time of this study and their carers.

### 2.2 Participants’ recruitment

Participants were recruited from the county of Essex using a purposive sampling approach. Local community-led friendly cafe groups (formerly known as dementia cafes) were identified, and then persons living with dementia and their carers were referred by the volunteers of the cafes/groups. An email was sent to the convener of the community-led friendly cafes and representatives of community groups, and following approval, the researchers visited the cafes to introduce the research project to the members of the cafes. A contact email address was collected for those interested in participating. Both the participant information form and consent form were sent over email ahead of the interview, and a convenient interview time was arranged after a 15-day ‘cool off’ period to consider their participation. Consent was taken again at the beginning of the interview. Recruitment was done over a period of 4 months between May and July 2025. The study included only those participants who had the capacity to consent.

### 2.3 The sample

The planned sample was eight persons living with dementia and eight carers, of whom two were persons living with dementia –carer pairs. A maximum of three attempts was made to arrange interviews. By the end of the study, eight carers, four persons living with dementia, and one participant, who was a carer as well as living with dementia, were interviewed, with one being a person living with dementia –carer pair. Interviews were carried out in an environment chosen by the participant, either in a private room associated with the community-led friendly cafes, in their own homes, in a private room at the University, or as an online interview. Only person living with dementia with a capacity to consent were recruited for this study. Carers were consulted to confirm the capacity of the person living with dementia to consent. Section 1(2) of The Mental Capacity Act (2005) states that “A person must be assumed to have capacity unless it is established that he lacks capacity.” Furthermore, following the guidance in sections 1(3) and 1(4), in those cases where the person living with dementia were not accompanied by a carer, the researchers engaged in an initial conversation to test their capacity to communicate and understand their basic comprehension levels. The participants were considered to have the capacity to consent, based on this initial screening.

### 2.4 Data collection

This study is guided by the social constructivist framework, which involves a collaborative, dialogue-based approach to generate socially derived perspectives of the issues experienced by the carers of persons living with dementia. Semi-structured interview guides (see Supplementary material S1), congruent with the social constructivist framework, were used to interview eight carers of persons living with dementia, four persons living with dementia, and one carer, who was themselves living with dementia. All interviews were recorded using Microsoft Teams to assist the transcription process. The interviews were then read by the researchers, checked for accuracy, and edited for corrections. The recordings were deleted after anonymising the edited transcripts. Each transcript was given a code, and the names of people and places were replaced with “[name]” and “[place],” respectively. The carers are referred to as Carer1 to Carer8, and persons living with dementia as P1 to P4, to ensure anonymity and to clearly distinguish the two categories of participants. The third category, where there is only one participant, who was living with dementia as well as a carer, is referred to as C + P 1.

### 2.5 Data analysis

Descriptive statistics were used to summarise the profiles of participants, and six step thematic analysis (Braun and Clarke, 2006) was utilised to extract the themes from the anonymised interview transcripts.

## 3 Results

The results are organised into: (1) persons living with dementia, (2) their carers, and (3) carers of persons living with dementia, who are themselves diagnosed with dementia.

### 3.1 Participants’ profile

The age of carers ranged from 60 to 87 years, and the number of years since the dementia diagnosis of their family members ranged from 2.5 to 12 years. All eight carers cared for their partners, though one carer cared for their partner as well as their adult child. Partners of three out of eight carers had Alzheimer’s disease, one had a mix of Alzheimer’ disease and vascular dementia, another one had frontotemporal dementia, and one had Lewy Body. One carer did not mention the type of dementia, and the other one mentioned early onset but not the type (Table 1).

**Table 1:** Participants’ profile.

Code	Role	Age	Dementia type	Relationship	Years post-diagnosis	Key context (Notes)
Carer1	Carer	74	Alzheimer’s Disease	Partner/Spouse	12	Long-term caregiving
Carer2	Carer	61	Mixed: (AD and Vascular)	Partner/Spouse	7	Young onset; diagnosed at age 55
Carer3	Carer	77	Early onset (Unspecified)	Partner/Spouse	NR*	
Carer4	Carer	60	FTD (Logopenic variant)	Partner/Spouse	5	
Carer5	Carer	64	Lewy Body	Partner/Spouse	2.5	
Carer6	Carer	87	Mixed (Son & Spouse)	Multi-Carer	Son: 10+; Partner: 5	Caring for two family members
Carer7	Carer	63	Alzheimer’s Disease	Partner/Spouse	4	Diagnosed during COVID-19.
Carer8	Carer	NR*	Alzheimer’s Disease	Partner/Spouse	3	
P1	PLwD	81	Frontotemporal	N/A	3	
P2	PLwD	78	No formal diagnosis-	N/A	NR*	Clinical Cognitive impairment.
P3	PLwD	81	Early onset (Unspecified)	N/A	NR*	Interviewed as a dyad with partner.
P4	PLwD	71	Lewy Body	N/A	6	
C + P 1	Dual	NR*	Vascular	Partner/Spouse	1	Discrepancy: Clinic vs. Doctor dx.

^*^NR = Not reported.

The age of persons living with dementia ranged from 71 to 81 years, and the number of years since their diagnosis ranged from 3 to 6 years. Three participants who were living with dementia received a formal diagnosis, one had not received a formal diagnosis (their self-identification allowed participation in this research), and one did not mention the number of years since diagnosis. Out of these three persons living with dementia who had received a formal diagnosis, one had frontotemporal dementia and one had Lewy Body (Table 1).

### 3.2 Thematic analysis of implications on carers

**Figure 1:**
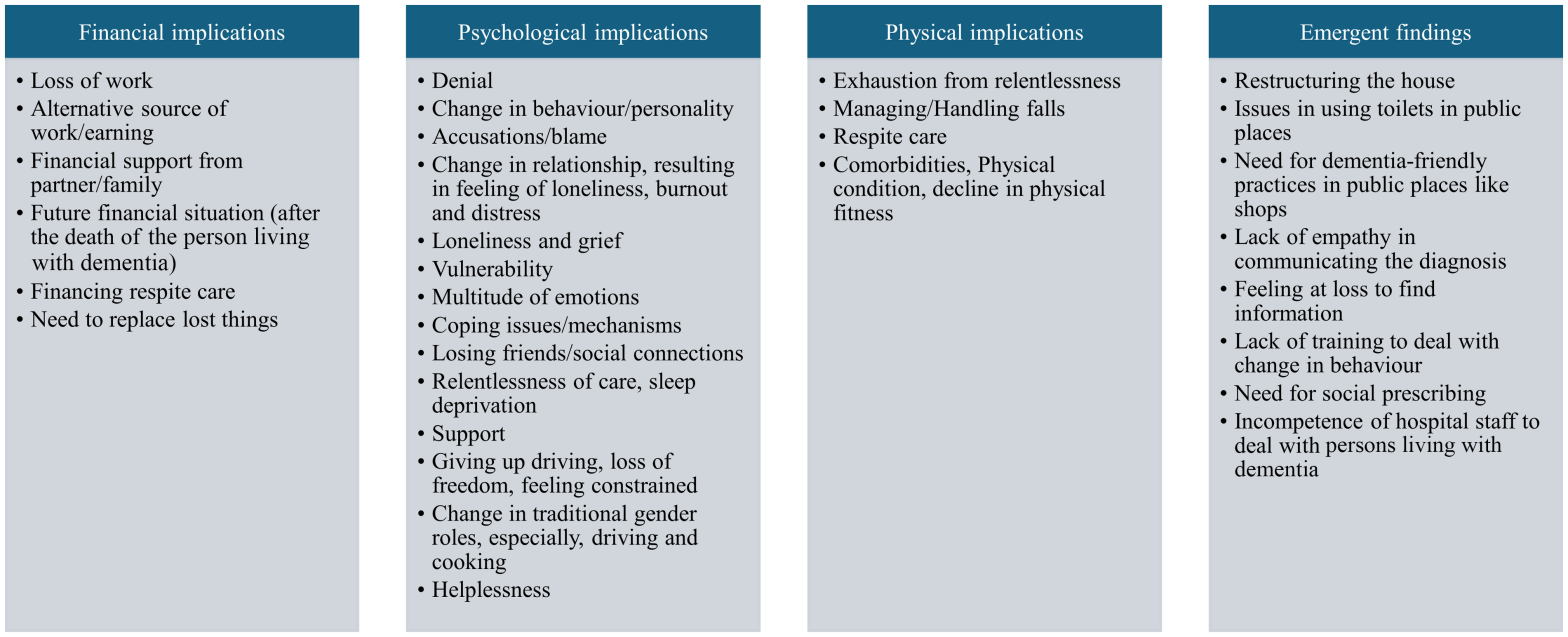
Thematic map: implications for carers of persons living with dementia.

Carers explained how the dementia diagnosis directly affected them, but also influenced the entire family and how it also caused fundamental changes in the relationships with other family members (Figure 1).

#### 3.2.1 Financial implications

The loss of work emerged as a dominant theme among the carers (Figure 1). This was either in the form of early retirement, largely arising from the necessity for increased care responsibilities, or in one case, because of their (carer’s) own ill health. One of the carers also mentioned alternative sources of work/income, starting their own business, working part-time, or even mortgaging their house.

Financial support from partners, or from other family members through inheritance, had also become a source of income for some carers.

Concerns about the financial situation in the future, especially after the death of the person living with dementia, were found to be a cause of concern and distress. One carer commented:

“*when the end comes, I do not actually know how they would value it [property] should they ever have to go for care? So, I do not know how that actually affects my money. I am worried about that because we have never married…*” (Carer3).

Another carer stated:

“*Yes, we had financial assessments… and I think we are on the borderline, you know. At this stage, we are paying some of the cost of the home.*” (Carer6).

Uncertain situations such as COVID-19 caused carers to express their concerns about financial uncertainties, augmented by insufficient information about financial support, in such unforeseen circumstances.

Even while caring for person living with dementia, carers are required to make arrangements for respite care. When other family members are not available for respite care, the financial burden is significant, with limited financial support available from the government. When family members were used for respite, carers also reported feelings of guilt in leaving the person living with dementia, whom they cared for, with family members to deal with.

Some carers, and indeed even the person living with dementia, reported that they tended to misplace items around the house and garden that are often never found again. The carers are required to spend money on buying those lost items. One example of the cost to replace lost items was given by a carer:

“*We did go through a stage where they had been very good at DIY and things, but all they could do at that point in time was to dismantle the machines. So, we went through three lawn mowers that got taken apart and three vacuum cleaners. So, I went back to a point where actually I had to hide things like that because I cannot afford to keep replacing them.*” (Carer1).

#### 3.2.2 Psychological implications

Psychological implications were reported significantly more than financial implications amongst carers.

Carers reported that the person living with dementia they cared for were in denial of diagnosis.

“*…[name] has been in denial for a while. But come round a little bit to the scenario of dementia.*” (Carer3)

“*… they will not actually accept that they have got it… they have always been into sport, they have always a winner. They have to win everything. They will not admit that their body is letting them down. They said nothing wrong with me.*” (Carer8)

Carers noticed a marked change in behaviour of the person living with dementia, often accusing the carers (and other family members) of taking away their independence. Carers also reported the change in behaviour of the persons living with dementia they cared for, in the form of agitation, lack of empathy, frustration, and lack of confidence (even before diagnosis and in many cases, intensified after diagnosis, with the progression of the condition).

“*The last 3–4 weeks, he has taken a massive dive though. Literally, they have been verbally abusive towards me.*” [sic] (Carer2)

“*There’s a loss of empathy, which is just such a massive impact from a person who’s not naturally empathetic anyway. To lose even more is just massive.*” (Carer4)

“*They cannot put the battery in the lawnmower… So sometimes they get frustrated.*” (Carer7)

“*… when they retired, they sort of seemed to have lost a lot of their confidence.*” [sic] (Carer8)

Carers expressed the feeling of loneliness arising from a fundamental change in their relationship with their partners, from bidirectional (partner) to unidirectional (carer) responsibilities.

“*You feel very alone. And it’s not just your personal relationship.*” (Carer1)

“*So I wake up every morning, that’s probably the thing I miss the most about it, is [name] getting up and making us both a cup of tea, but it’s simple.*” (Carer2)

Loneliness was found to be augmented with grief of losing a loved one, bit-by-bit each day, or seeing a complete change in their personality or behaviour:

“*… life has changed and [since] their diagnosis because the first thing that hits you when they are diagnosed, and I think this hits the carer more than the person, is that it’s sort of a bereavement that hits in…*” (Carer1)

One of the carers expressed that this change in behaviour has resulted in the carer developing an dislike towards their partner.

“*I did not like them. And we have been married for 30 years. They were vile, you know, they’d say horrible things.*” (Carer2)

The feeling of vulnerability was strongly expressed by one of the carers:

“*… my fear is that if I, my health goes down, they cannot even call for help, … that’s where you start to think, and that’s why mainly we moved. So that’s when you start to feel incredibly vulnerable.*” [sic] (Carer1)

On the other hand, one carer narrated how their partner, a person living with dementia, continued to be empathetic, expressing their concern about the carer’s happiness. This empathy, in fact, was distressful to the carer, since the person living with dementia wanted their partner to leave them to live a happy life.

“*Yeah, the most distressing thing… they sometimes know how unhappy I am. And just over a year ago,… they said I will leave [name]. [Repeats] They would leave me so I could be happy.*” (Carer7)

In the above quote, the carer subtly expressed that the caring responsibilities, at times, make them feel unhappy.

Issues coping with the condition, the multitude of emotions, as described above in this section, had been difficult for some carers, ranging from explicitly mentioning they were not being able to cope to others talking through the mechanisms to cope with the emotional burden:

“*… it’s probably been about the last year, I’ve been thinking, I do not think I can cope much more.*” (Carer2)

“*… so, I looked into what to expect, and I went to carers groups quite quickly and the information groups that Alzheimer’s did at that time… I had to admit I was horrified when I went to the care groups because people were talking about their everyday situations, and it’s beyond your comprehension at that point in time. I do not know whether that was a good thing or a bad thing to go [there], but it certainly gave me an insight into what I was really facing. I think in the long run, it did good [to me] because I then thought, it’s not just them being difficult… this happens to [all who have] their person/loved one [with Alzheimer’s/dementia].*” [sic] (Carer1)

Many carers expressed the pain of losing their friends and social connections after their partners were diagnosed with dementia. Friends just stopped visiting them.

“*They do have a few good friends, [from work] and they have stayed…, but generally we have lost a lot of social friends.*” (Carer1)

“*We have had some friends who were very good friends of ours once they found out about the diagnosis, just never heard from them.*” (Carer5)

Relentlessness of care also emerged as a major theme and was expressed by several carers. This is evidenced by care being a 24×7 duty, changing the sleep cycles to match the changes in that of the person living with dementia, or keeping an eye on them remotely, even while at work:

“*… it’s basically if everything is on me, literally financial, washing, shopping, ironing, food… I’m literally living this 24/7. I mean, even at night now, you know, I’ve got to [support] while they pee… and unfortunately, they are waking up early, which means I’m absolutely exhausted. I mean, they started sort of 3:15 a.m. waking up.*” [sic] (Carer2)

“*… You know, and we had the ring doorbell, so I could sort of see what was going on. And when you are at work, and your partner is trying to get through the door. But they cannot find how to work the key, and they lost the key, and they were climbing on the bin over the fence. So I’m seeing all these on the ring doorbell at work. Yeah, so off I go…*” [sic] (Carer7)

On the other hand, a lot of carers expressed the support they have received from the support groups, such as friendly cafes. They also expressed that the change in name from “dementia cafe” to “friendly cafe” is a positive change, as it helped them change the perception that these cafes do not have only persons living with dementia. However, changes in services offered by friendly cafes because of uncertain funding resulted in confusion and a lack of dependability to carers.

“*I did not know where to go because the funding changes and then, people just disappear. So [in the first], you think that you know where to go and [then,] they are not there anymore.*” (Carer1)

“*The [organisation] came in and … I’ve been told that they got a grant from [name of the] Council to do that training [for handling falls]… I never heard anything. Never got it. So I do not know what happened there, could be that the funding was withdrawn.”* [sic] (Carer4)

Another major issue identified was the reluctance to give up driving, and the traditional role of men in driving, making it difficult for their partners (who are also their carers) to begin driving. One carer expressed:

“*… the only thing they get distressed with is that I will not let them drive now… I tell them it’s because of their medications for his prostate cancer, because they will not accept that we do not want him driving. They’ll say how long until I’ll be driving? And I say well, you are off this medication.*” [sic] (Carer8)

Another carer reported on a conversation they had:

“*So plucked up the courage and I just said to them, I do not think you are safe driving… So, a few weeks ago, their forms came through to renew their licence. I’ve got the forms, and I put them on the mantelpiece, and I said these are the forms to renew your licence. I will leave them here. You can decide what you want to do, but I think you should give up. You’ve got points on your licence from last year, you were stopped by the police.*” [sic] (Carer4)

Changing roles, such as taking up driving, may be a source of stress for carers who have not done so before:

“*The stress on me is I have to drive everywhere, you know, even if we go to a garden centre for a cup of tea. But taking them out makes them happy. But for me it takes its toll.*” (Carer7)

A carer of one person living with dementia expressed the grief of losing their partner, at first to a care home, and the feelings of helplessness when wanting to support them but having to do this reciprocally with the care home staff. The carer expressed feeling less helpless through being able to support their partner, whilst in the care setting.

“*… the carers [from the care home/care home staff], where they are now, say to me, ‘if you can do this’, [or] ‘if you can come and help’ or that ‘would you mind [doing]that’… I could feel that I was doing something…*” (Carer6)

#### 3.2.3 Physical implications

Relentlessness of care was found to have psychological as well as physical implications on carers. The psychological implications highlighted the relentlessness, and the physical implications of relentlessness were also experienced by the carers in the form of fatigue and exhaustion. As expressed by some carers:

“*… I’m actually exhausted, [physically] and mentally. I’m absolutely drained. I’m, from the minute I get up, I’m focused on them… Doing errands, paying bills, you know, things like that, you know, but it’s physically exhausting, and there’s not enough time for carers.*” [sic] (Carer2)

Moreover, carers also expressed the difficulty in extending physical support to their partners, also because of their own age and issues such as arthritis (one carer reported having it). For example, some carers were required to provide support to the person living with dementia, they care for, when they wanted to stand up from a sitting position (from a chair, bed, couch, etc.), and getting in and out of a car. Carers are also required to physically support the person living with dementia to stand up after a fall.

“*Even when they stand up now, I sometimes have to get caught for help… I’ve only just started to have carers for them, for their personal care because I simply cannot manage him*.” (Carer1)

“*I’ve had to find a device that helps [them] get in and out of a car.*” (Carer5).

“*… they kept having falls. We had the paramedics out because we were advised not to try and lift… The paramedics came out three or four times, and of course, they do not come immediately… They would not/could not seem to direct their limbs to do… for instance, if they fell in the bathroom and tried to get back… to bed. I said ‘No, stay there’ [but it is] difficult when it’s four or five hours to keep somebody there… I’m finding that I’m not as fit as I was… I’m not able to do that part from the fact that I’m getting older.*” (Carer6)

“*before they went into hospital [for pneumonia], they did have a fall and had to be lifted, and also I’ve got arthritis in my left arm…*”[sic] (Carer8)

This also calls for the need for respite care, and challenges associated with it, a theme that clearly emerged from the interviews:

“*… my middle son, he lives in [place] and when he comes down, perhaps once a week, when he can… he does my Internet, and computer work and all that… which is great help.*” (Carer6)

However, most carers found difficulties in working out respite care:

“*I had to think how that would actually inflict on their [family carers other than the partner/main carer’s] lives as well.*” (Carer3)

“*That’s fine, but they [the person living with dementia] do not want to get involved in care plans… It’s not a problem because… I’m currently talking to the middle daughter, but suddenly she disappeared last year.*” (Carer4)

Carers also mentioned having some support in place for the person living with dementia, for physical tasks such as helping them get in and out of the car. Alongside all these ‘in the moment’ examples of physical implications, carers also had comorbidities such as diabetes, hypertension, and prostate cancer (surgery), resulting in a decline in physical fitness.

#### 3.2.4 Emergent findings

Apart from the financial, psychological, and physical implications, a few more issues also emerged:

Carers discussed the requirement to make changes to their houses:

“*So we have the back to the wall, toilets put in at 19 inches. We had a wet room, big enough that they could sit on or I could sit on this chair, and then come out and have a chair outside and dry. So we have actually future-proofed.*” [sic] (Carer3)

“*… there’s stiffness, mobility, and hallucinations, and so for mobility, it’s been getting things like the walker in place, having the bed with the [railing] because they were falling out of bed… We had to have the shower changed because again, we are tripping and they cannot step getting off the toilet. So, there’s been a lot of changes that have had to happen to make life easier for them, but I think trip hazards, you just would not believe the things that you would trip upon, until you are living with someone.*” (Carer5)

Carer highlighted, for example, the complications of using toilets in public places, where each toilet has different types of taps and soap dispensers, which have resulted in person living with dementia getting confused on how to use it. One carer strongly emphasised the size of the toilets on the flights. The persons living with dementia might need support from the carer to get in and use the toilet. However, there is hardly any room for one person to get into the toilet.

“*… you are going to the toilet on a plane… they [the person living with dementia] do not know that you need to put the thing across. So, obviously, I’ll go with them, and on some planes, you cannot get two people in, you know? So I’m waiting outside…*” [sic] (Carer7)

Carers also expressed the need to implement dementia-friendly practices in public places. For example:

“*And we went in and out of shops… Things like the mats, doors opening easily or not. Lights. Music… It sort of blocks the thinking… Layouts of the shop, where it’s quite confusing sometimes, they have got too many islands in, it is not clear where you are. We checked, and it is the exit sign. Was there the toilet sign? We did not, you know?*” [sic] (Carer1)

Another carer narrated about sudden shouting or people being very loud while talking, while yet another expressed the confusion arising from the supermarket staff talking over the headsets.

*“In supermarkets, they talk to each other (on headsets). I have to say stop, they have got dementia, because they [the person living with dementia] think you are talking to them.*” (Carer2)

“*You know it’s the hole [interpreting black-coloured doormats as a hole] thing… and if it [the doormat] has too much detail [fine designs] that is also confusing, but the dark mass has to be lighter. Name tags are important: you have got [to have] big [fonts] because if my partner [is trying to] relate or answer their questions, it is so much easier if I say, could [name of the person living with dementia] talk to you?*” [sic] (Carer3)

Carers expressed the general lack of empathy while communicating the diagnosis of dementia, and also experienced a lack of coordination between the general practitioners (GPs) and the memory clinic:

“*… the GP’s on the medical side are good if there’s a medical problem… they monitor their [the person living with dementia’s] blood sugars, but the second you wanna talk about the dementia… [gestured as, they do not want to]… He’s not had a review with a dementia specialist since his diagnosis, because it cannot be treated with medication, you treat with management.*” (Carer4)

“*The thing is, when they give you that diagnosis, it turns your whole world upside down because we had a lady come in from the [clinic name] memory clinic. And the first thing she said to us was, have you got your lasting power of attorney in order?. And we are going to do this test. It was all very there was no sort of understanding; there was no empathy.*” (Carer7)

Carers also expressed their concerns about not having the information on where to get the information that would be helpful to carers of people newly diagnosed with dementia.

“*I did not even know that you could have this grant for… the carers and you get it once a year from the [name of the] Council.*” (Carer5)

“*… the Universal Credit… Three years before I found out [we were eligible].*” (Carer2)

“*All of my information from [is from] TikTok and YouTube.*” (Carer4)

Lack of training to deal with behavioural changes resulting from dementia, handling delirium, and manual handling of the person were found to be additional challenges of caring for the person living with dementia.

“*There’s no training. You’re just left [with someone having a] challenging behaviour, it’s difficult, and I’ve had to remain calm under very difficult conditions. With no training, I just stood there entirely reliant on how well I know that person… they are prone to delirium as well, and you are left to sort out all these mental health problems with no training.*” (Carer4)

The need for social prescribers, and the support received from social prescribers and care agencies, was also highlighted in the interviews.

“*… It’s almost like a dark area… [The social prescriber] directed us for Council tax reduction,… a mobility scooter because… for all the other things - toilets, showers, the wet rooms, buying the extra cooking equipment, we paid for all of that.*” (Carer3)

One carer narrated their experience of their partner, who was admitted to Accident & Emergency (A&E), and the incompetence of the staff to deal with patients who are persons living with dementia.

“*At the A&E, they [the staff] did not take their [person living with dementia’s] notes very well… they [the person living with dementia] were allowed to dehydrate again and again… it was too hot. I had to ask three times to get a referral to the dementia nurse… it turned out she was off on long-term sickness… they [the person living with dementia] still got neglected because the type of delirium they have is the quiet type.*” (Carer4)

### 3.3 Thematic analysis of implications on persons living with dementia

**Figure 2:**
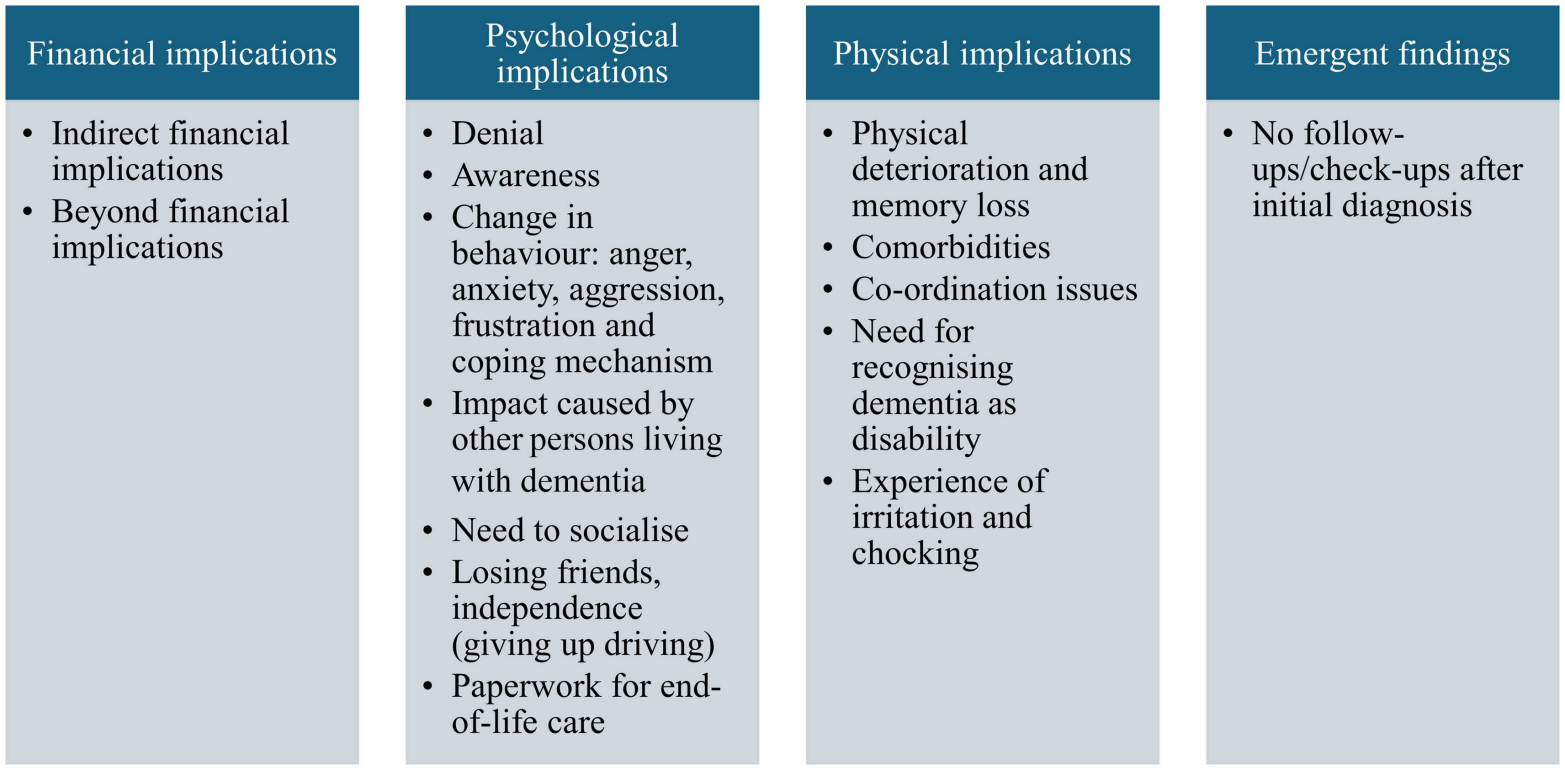
Thematic map: implications of dementia on persons living with dementia.

Persons living with dementia expressed more about their involvement in activities and that volunteering helped manage their behavioural changes resulting from the diagnosis, also that awareness about being diagnosed with dementia helped them chart their actions to manage the condition (Figure 2).

#### 3.3.1 Financial implications

Persons living with dementia hardly spoke about the financial implications. One of the four participants, who was a Person living with dementia except, mentioned that while his employment was not affected because they were diagnosed with dementia after his retirement, they did incur expenses on insurance for their back surgery, and said that there are created indirect financial implications.

“*It did not because… I was retired… it’s just that I had a back operation and insurance went up to [a huge amount] and … they [insurance] will not pay us back, so I suppose… it affects you financially as well.*” (P4)

Another participant repeated the phrase:“*… things aren’t all about money*” (P3) on a number of occasions during the interview.

#### 3.3.2 Psychological implications

Denial of the diagnosis was explicitly expressed, though one of the participants believed that they had dementia, despite not having a formal diagnosis.

Some participants showed awareness regarding dementia, the importance of diagnosis, and the importance of having an understanding of the condition; one of them subtly mentioned the stigma associated with dementia.

“*That is because we know in some cases there is treatment. In other cases, there’s no treatment, and in some cases it’s rapid. And in some cases it seems to settle down, and you do not get any worse… in most cases, I think, it does get worse… depending upon the diet, whether it’s vascular, Alzheimer’s or whatever… the diagnosis helps, it gives you a working understanding.*” [sic] (P1)

“*I want to see it. So I’ve got a disc, and I’ve got a photograph of it, and he showed me the plaques that are growing in the brain cells at very, very early stages… Lewy body, they [the doctor] said, this is plaques growing in your brain cells. They might grow. They might stay. They’re never going to go away. So that will eventually fill up your brain cells.*” (P4)

“*I think a lot of people think there’s some kind of stigma… and therefore they do not talk about it and that’s no good.*” (P2)

Participants discussed changes in their behaviours, such as getting angrier than earlier or experiencing frustration, and the coping mechanisms adopted by them to keep a check on anger and aggression.

“… I*’ve always had a temper, but always controlled it and… I was beginning to lose my temper more so. I tend to throw things when I really lose it, and so from that moment onwards I became much more aware… I then concentrated on what I thought might be things that would help in any way … In some ways, my life became more positive because… I’ve sort of tried to keep my brain more active, so whereas the hands were active [earlier], the brain’s more active now.*” (P1)

“*I find it very frustrating if I meet that person, maybe in a couple of days’ time… I just cannot remember them, and it gets a little bit sort of awkward at times… I find that really frustrating and… if we have been talking about something and then the conversation may stop, then return shortly after that, I just cannot remember what was said.*” [sic] (P2)

“*… I had lost confidence [in driving]… but anyway, now I’m out there driving. I do art. I have never done art in my life and I do… I taught myself… and I’ve sold some… and the money has gone to the dementia group in [place]. I wanted to start [cooking]. I wanted to make my daughter her 40th birthday cake, and I did. It took me time, but I’ve done it, and it turned out very, very nice, and it looked lovely. So it’s a matter of not giving up.*” [sic] (P4)

Persons living with dementia also discussed the experience of distress caused by other persons living with dementia.

“*… And that’s when I was volunteering in another local organisation, I used to take a gentleman out, and he’d have a bit of lunch. He had frontal temporal dementia, quite advanced, and one of those symptoms is you might not be able to swallow, and one day, when I was taking him out to lunch, he choked on his food… it’s distressing to see how rapid it is and the initial awareness, and then they cease to be aware of what’s happening to them.*” [sic] (P1)

“*… they do not want to be sitting around not being horrible here, but they do not want to be sitting in a dementia group with like 80 or 90-year-olds. People who are quite advanced. They do not need that. I do not need that. I’m not at that stage yet.*” [sic] (P4)

Losing friends after diagnosis and the need to socialise and be around people were expressed by persons living with dementia:

“*It’s just that word dementia, as soon as you mention it, friends, first of all disappear…*” (P4)

“*I just thought you are having dementia and you go to clubs and you meet other people and you blend in and it’s nice to be around people.*” (P3)

The hassles experienced for getting the driving licence renewed and loss of independence by having to give up driving were also mentioned by one of the persons living with dementia.

“*I asked for it [memory test] because I have to have my driving licence checked every year now… I only get a yearly licence… and they [memory clinic] said they are not going to give you any more memory tests… well, I have to give this evidence to the DVLA, to the driving people. So I particularly went to the doctors… I’ve got one letter wrong [in the memory test]. So I’ve got 29 out of 30, which is still good, and I’m still waiting for my driving licence to come back… I will need my driving licence because this is where I get stressed now, because I’m doing a lot of voluntary work.*” (P4)

However, there was concern about the timing of the paperwork for end-of-life care, as too early would risk denial, and too late may result in a lack of ability to consider things rationally, as could be gauged from the following quote from a person living with dementia. They do not want to fill in the end-of-life care form at this stage, and do not want to be a burden to the family when the time comes.

“*I’m not going to be a burden on my family either… when the time comes… they give you this form…‘this is me and what do you want to do when you have got… If you have got a care?’ I ain’t filling none of that in and do not resuscitate… because I’m not going anywhere.*” (P4)

#### 3.3.3 Physical implications

Physical deterioration and memory loss with progression of dementia emerged strongly in the conversation with persons living with dementia.

“*… in most cases, I think it does get worse. Depending upon the type, whether it’s vascular, Alzheimer’s, or whatever.*” (P1)

“*Maybe not two or three days later, and they say, well, if you remember, I told you that, you know, a couple of days ago, and you just cannot remember.*” (P2)

Other physical implications included comorbidities (heart attack, fibromyalgia, osteoporosis, sleep apnea, and its treatment—continuous positive airway pressure—CPAP) and coordination issues.

“*It [co-ordination] might have been [affected], but I cannot remember.*” (P2)

One of the persons living with dementia expressed that they have been registered as “disabled” for other disabilities, not because of dementia.

One persons living with dementia also mentioned occasionally experiencing irritation and a choking sensation on the left-hand side.

#### 3.3.4 Emergent findings

Lack of follow-up/medical advice after diagnosis has been a concern for all the participants formally diagnosed with dementia. They also felt a lack of support from the system, for example, one participant said:

“*… after your initial diagnosis, no more check-ups. And, and I know the resource is not there, but I think an awful lot of people would be reassured if there was an occasional check-up.*” (P1)

Another narrated her conversation with the GP surgery:

“*Well, can I have another brain scan? No, you cannot.*” (P4)

### 3.4 A carer of a person living with dementia, who were themselves diagnosed with dementia

One participant in the sample was a person living with dementia, themselves and was a carer of their partner, also a person living with dementia. This case has been analysed separately, as it would give insights into how different the struggles faced by someone who has the condition, as well as being a carer, than just being either of these.

#### 3.4.1 Financial implications

Since the participant was already retired at the time of dementia diagnosis of her husband, there was apparently no impact on earnings. However, she expressed concerns about receiving an attendance allowance (perhaps, for attending friendly cafes, most of which are themselves dependent on available funding), which had apparently stopped, and that they should be entitled to a waiver of council tax, which implies the financial constraints that they might be experiencing.

#### 3.4.2 Psychological implications

The approach of the doctor, when conveying the diagnosis, can have a strong psychological impact. She reported:

“*The doctor was quite crude about it, really. Just said you have got vascular dementia, and when your head’s full of cholesterol, you’ll be dead. And I walked out of there and said, what? Well, I’ve got to die of something*.” (C + P 1)

While talking about her husband’s diagnosis, it was more of a denial on her husband’s part.

“*Do not think I had the one [name] did okay, and that’s when they [the person living with dementia] told me they were perfectly all right, because they got all their questions right [in the memory test], [whereas] in fact, [the person living with dementia] actually had multiple [transient ischemic attacks] TIAs.*” (C + P 1)

They expressed the loss of independence because of the inability to drive (which included surrendering the driving licence). However, they had switched over to public transport (bus and train) and kept them mobile.

“*We still go out, and perhaps not as much as we used to do, but it’s not quite so easy…*” (C + P 1)

Talking about their partner [a person living with dementia], they narrated the change in character, especially anger and blame.

#### 3.4.3 Physical implications

The participant themselves was a prediabetic, and they had to take care of their own sugar levels. They also experienced frailty. This compounds the burden of caring for one’s own self for comorbidities besides dementia, while simultaneously caring for their partner, also a person living with dementia, despite receiving respite care from their son.

#### 3.4.4 Emergent findings

Just like other participants, they too had difficulty finding information about the support and benefits that a person living with dementia and their carer are entitled to.

## 4 Discussion

Existing literature has largely examined this problem in silos, without accounting for the interconnections between financial, psychological, and physical implications as well as the combined experiences of both, the person living with dementia and the carers. While there is some evidence on the psychological and physical issues experienced by the carers of persons living with dementia, there is no discussion of the financial issues experienced by these carers. Our study has provided new evidence on the interconnection between financial issues experienced by the carers of persons living with dementia (both with and without comorbidities), over and above psychological and physical issues experienced by both the persons living with dementia and their carers. Here, we explore the impact of these three issues, showing how, for example, financial constraints may limit access to respite care, which may, in turn, intensify the relentlessness of care, while increasing the physical burden.

Literature on financial implications of dementia on families not only includes loss of wages due to early retirement or reduction in working hours by both, the persons living with dementia and their carers (Nandi et al., 2024; Bayly et al., 2021) but also in terms of expenses for prescription, inpatient and outpatient care, which multiplies for those with comorbidities (Coe et al., 2018). This literature is largely in the context of the USA, which has a healthcare system financed by social insurance, as compared to that in the UK, which is financed by the government. Despite this difference, the implication in terms of early retirement and reduction in working hours is elicited from our study too. There is a dearth of literature on financial distress experienced by the family unit, as a whole, and by the carer, though studies show that families incur 70% of the total cost of care, and the costs of caring for dementia is 86% higher than those for those without it (Jutkowitz et al., 2017). Carers were also found to express the need for in-depth planning for dementia care and allocating a separate basket of funds exclusively for care expenses (DaDalt et al., 2016). The need for in-depth planning is supported by our study too, where carers were found to generate finances through mortgaging their property, while some were fortunate to have support through inheritance. On the other hand, those who were even partly dependent on the financial support from the government were concerned about sustaining themselves as the support would stop after the death of the person living with dementia and the long career break augmented with age, would make it difficult for the carer to find work afresh, which requires the plans to provide for the sources of finance, after the death of the person living with dementia. Another most common cost component involved is the structural modifications to the house, which is the bathroom safety and modifications focusing on improving mobility, safety, accessibility, and falls prevention (Marquardt et al., 2011; Cha, 2025). However, this has also been found to be highly cost-effective (Cha, 2025).

Beyond financial distress, carers experience multiple psychological issues. Denial has emerged as the most common issue experienced by both the person diagnosed with dementia, as well as the carer, and this has been one of the barriers for clinicians in communicating the diagnosis (Milby et al., 2017; Wollney et al., 2022; Parker et al., 2020). Our study also found denial and coldness in communicating the diagnosis, albeit independent of each other.

One of the fallouts of denial experienced by carers has been the difficulty in convincing the person living with dementia to stop driving/giving up the driving licence, as per our study. Existing literature also found that family members have consistently, and over the years, experienced difficulties in convincing the person living with dementia to stop driving (O’Neill et al., 1992; Stasiulis et al., 2020). Studies have found that people with moderate and advanced dementia are at a higher risk of motor vehicle accidents (Lee and Molnar, 2017; Toepper and Falkenstein, 2019; Ott and Daiello, 2010), and that there needs a system in place whereby the Driving and Vehicle License Agency be notified of all new cases and the doctor should make a decision on whether the person diagnosed with dementia should continue driving or not (Breen et al., 2007).

Existing literature had identified that behavioural and psychological disturbance symptoms (BPDS), such as delusions, hallucinations, depressive symptoms, anxiety, euphoria, agitation, aggression, apathy, irritability, and disinhibition, were strongly associated with reduced quality of life for the person living with dementia as well as their carer (Banerjee et al., 2006; Borsje et al., 2018; Gaugler et al., 2009; Tan et al., 2005), also for changes in relationship of the carer with the persons living with dementia (Feast et al., 2016) and loss of independence and agency (Macquarrie, 2005; O’Rourke et al., 2015; Stumpf et al., 2025). Moreover, psychological inflexibility of persons living with dementia was found to be associated with anxiety and depression in carers (Kishita et al., 2020). Our study supports these findings of experiencing living with a completely different person than the one they knew, because of the BPDS, and in some cases, even developing a dislike for the person cared for, while experiencing grief in most cases.

The loss of partnership/relationship and loneliness experienced by the carers of persons living with dementia, as elicited from our study, is also supported by the existing literature (Kotwal et al., 2024; Kovaleva Kovaleva et al., 2018; Victor et al., 2021; Ryder, 2016). Studies show that carers dedicate all of their time caring for the person living with dementia, resulting in overburdening themselves (Leite et al., 2017). Care responsibilities get more demanding with the progression of dementia, resulting in psychosocial vulnerability, manifesting in the form of grief, fear, and feeling trapped (Cormican et al., 2022). The need for respite care arises from the relentlessness of caring, though carers were found to experience guilt when they are away from their care duties (Gaugler et al., 2009; Prunty and Foli, 2019; Romero-Moreno et al., 2014); these observations are supported by our study too.

Many studies have examined and identified emotional exhaustion, depression, anxiety, as well as burnout among the carers of persons living with dementia (Alves et al., 2019; Campos-Puente et al., 2019; Gratao et al., 2010), with some looking at the interventions to deal with stress and depression experienced by the carers, from a clinical as well as non-clinical perspective (Cheng et al., 2019; Felstead et al., 2023; Leung et al., 2023; Li et al., 2012). Our study supports the literature that community-led friendly cafes and community groups have given carers a safe space for socialising, to deal with isolation, and a respite from the relentlessness of caring.

Existing literature has found that carers experience sleep disturbance and have a poor sleep quality (Song and Kim, 2021; Chiu et al., 2014; Ali and Bokharey, 2015). Physical exhaustion/fatigue was also found to be common among carers (Gratao et al., 2010; Lee et al., 2025; Ali and Bokharey, 2015), and supported by our study.

Literature further shows that musculoskeletal disorders are common among the carers, in general (Wyton, 2018), and physical discomfort is experienced by the carers while manually handling the persons living with dementia for their transfers in and out of the toilet, shower/tub, car, chair, or picking them up from falls/holding them to prevent falls (Darragh et al., 2015). On the other hand, there is a need for increasing the awareness among the carers regarding falls prevention (Mamani et al., 2019; Thomas et al., 2016), and that carers’ awareness and right attitude regarding the risk of falls and their ability to communicate these risks with the persons living with dementia can contribute to falls prevention (Ang et al., 2019). There is a need to develop education/training programmes for carers on manual handling (James et al., 2017). Our study highlighted that carers are not equipped or trained, and experience difficulties handling falls in particular, and for their ADL, in general.

Physical health issues experienced by persons living with dementia differ to a great extent from those experienced by the carers (Bom et al., 2019). Moreover, studies over the years have shown that carers of persons living with dementia experience physical health issues, which are largely stress-induced (Fonareva and Oken, 2014; Schulz et al., 1995; Pinquart and Sörensen, 2007). Carers were found to have higher cortisol levels (Richardson et al., 2013) and specifically, the African-American carers of persons living with dementia were consistently found to experience a higher diastolic blood pressure (Knight et al., 2007). Young carers, on the other hand, were found to experience headache, tiredness, back pain, and chronic mobility issues, a disability, over and above sleep deprivation (Lacey et al., 2022). Our study found physical exhaustion, to the extent of feeling drained, augmented by age-specific conditions such as arthritis. Physical health issues of persons living with dementia include physical deterioration, memory loss, and impaired coordination, though the carers were mainly finding it difficult to physically support the persons living with dementia in case of falls.

Assisting the person with the use of the toilet, especially in unfamiliar environments such as public places and air planes have been highlighted (Murphy et al., 2021). Absence of unisex toilets, or toilets clearly labelled for specific sexes pose difficulty in assisting the person living with dementia when the sex of the carer is different from that of the person living with dementia. The size of the cubicles often reduces the ability for the carer to assist, as is the case with toilets in airplanes, and can lead to the person living with dementia experiencing more distress as they can experience up to four times as many incontinence issues (Grant et al., 2013). Our study also found difficulties experienced by the carers in supporting the person living with dementia to use toilets in unfamiliar environments and airplanes.

Carers reported that when entering shops, cafes, or public spaces, the entrances laid out can be disorientating for the person living with dementia, and the use of Black doormats, bright lights, excessive noise or loud music, and staff communicating on headsets all contributed to the person living with dementia becoming more distressed or uncomfortable as persons living with dementia experience more visuospatial perception issues (Prince et al., 2011), which is supported by our study too.

In the two examples given above, the lack of any clear signs or signals that the person living with dementia may require additional support led to carers feeling they were constantly required to make people aware of the symptoms to ensure they were not misread in public spaces (Phinney et al., 2002). Carers felt that, in general, society and areas such as hospitality and retail lack awareness of dementia care and dementia in general, and this adds to the anxiety and distress experienced by them and the person living with dementia. It is this lack of awareness and support that carers also said they often felt from medical professionals. This was more often felt when contact with medical services was spread far apart, inconsistent, or created frustration when the services did not seem to have awareness about the issues experienced by persons living with dementia. It also included aspects of treatment that may not be immediately in connection to the dementia diagnosis, but that the person living with dementia requires an approach to care which is considerate of their needs (Watkins et al., 2019).

Consistent with our findings, existing literature suggests that the agency of carers is completely dependent on their own resources and skills for finding information, for navigating dementia care. However, the GPs, local health providers, and dementia groups, such as friendly cafes, also provide support in information dissemination (Leone et al., 2025; Innes et al., 2022; Charbonneau and Rathnam, 2020; Greenwood et al., 2017).

Literature has started to emerge on the benefits of the wearables, smartphone systems, and apps for persons living with dementia and carers, as well as for the self-management by the persons living with dementia themselves (Cornelius et al., 2025). Our study identified a case where the carer was able to trace the person living with dementia because of a wearable, and another case where the carer kept an eye, through smart home systems, on the movement of the person living with dementia while at work.

Our study has limitations. Our study includes people of only White ethnicity. The attrition in our study was relatively high. While several people (persons living with dementia and carers) committed to participating, attendance at the interview was inconsistent. Volunteers in the friendly cafe would report that members were unwell or had simply forgotten to attend that week. As a result, our participants (specifically persons living with dementia) were from a relatively healthier group. Our study does not include young carers, who may experience different types of issues and barriers from those explored and discussed here.

## 5 Conclusion

Programmes on care for carers would benefit from an integrated approach, addressing each of these issues in totality, given their interdependence.

Information about financial support available from the Government needs to be widely disseminated. Friendly cafes, GP surgeries, and memory clinics could be the primary centres for disseminating this information. People should also be guided to do diligent financial planning, providing for a chronic/long-term condition/illness, at an early age.

Friendly cafes should be made sustainable, with a provision for necessary support/counselling to deal with isolation, anxiety, depression, guilt, and other psychological issues experienced after diagnosis and arising from care responsibilities. GP surgeries and memory clinics are required to develop an empathetic approach towards people diagnosed with dementia and offer follow-up sessions to address the emerging issues with the progression of the condition.

Training is required to be provided to the carer to deal with changes in behaviour, physically supporting the person living with dementia for ADLs, as well as falls. Awareness regarding falls and precautions for falls prevention should also be a part of their training. Persons living with dementia should also be cautioned from time-to-time by their carer about precautions to avoid falls.

Public places are required to adopt a dementia-friendly approach. This could be done by adapting to changes in the colour of the carpets, bigger name plates, clearly labelled toilet signs, and future flight designs may consider designing dementia-friendly toilets. Persons living with dementia may be considered eligible for a Blue Badge too, because they live with subtle disabilities such as cognitive impairment and memory loss.

An integrated approach to post-diagnostic support for information dissemination, counselling, and training for carers could help reduce their care burden and provide more effective care for persons living with dementia. More research is required to be undertaken to understand the needs of persons living with dementia, which in turn can reduce carers’ burden.

## Data Availability

The raw data supporting the conclusions of this article will be made available by the authors, without undue reservation.
