# Supplementary material for: What needs to be addressed in caring for people living with dementia? A multi-faceted evidence on financial, psychological, and physical health issues in dementia care
Source: Front Dement. 2026 May 1;5:1790741. doi: 10.3389/frdem.2026.1790741 (PMC13175808; doi:10.3389/frdem.2026.1790741)
Supplement: Supplementary file 1 [file Data_Sheet_1.docx]

**Title of the Study:** People Living with Dementia and Carers: Financial, Psychological and Physical Health Implications

**Semi-Structured Interview Guide for carers of people living with dementia**

1. What is your age and since how many years have you been caring for your family member living with dementia? Has your life changed/altered since diagnosis? If so, how?

2. Could you give me an example of how your dementia diagnosis affected your work / employment?

3. Could you give me an example of how your life / lifestyle changed after being diagnosed with dementia?

4. Do you experience aspects of distress since you started caring for your loved one with dementia? If so, can you explain what this is and how it makes you feel?

5. Are you having any diagnosed health issues?

6. What effect have you experienced on your physical health since you began your care responsibilities?

7. Are there any other parts of living with dementia you feel are under-represented?

**Title of the Study:** People Living with Dementia and Carers: Financial, Psychological and Physical Health Implications

**Semi-Structured Interview Guide for people living with dementia**

1. What is your age and how many years are you living with dementia? Has your life changed/altered since diagnosis? If so, how?

2. Could you give me an example of how your dementia diagnosis affected your work / employment?

3. Could you give me an example of how your life / lifestyle changed after being diagnosed with dementia?

4. Do you experience aspects of distress since you were diagnosed with dementia? If so, can you explain what this is and how it makes you feel?

5. Are you having any other health issues other than Dementia?

6. Are there any other parts of living with dementia you feel are under-represented?
